# COVID-19 slightly reduced family resilience, coping, and disaster preparedness in ISTIFAR’s Lombok study

**DOI:** 10.4102/jamba.v16i1.1696

**Published:** 2024-10-22

**Authors:** Sriyono Sriyono, Hakim Zulkarnain, Jujuk Proboningsih, Kiki A. Kurnia

**Affiliations:** 1 Faculty of Nursing, Universitas Airlangga, Surabaya, Indonesia; 2 Department of Nursing, Politeknik Kesehatan Kementerian Kesehatan Surabaya, Surabaya, Indonesia; 3 Department of Chemical Engineering, Faculty of Industrial Technology, Institut Teknologi Bandung, Bandung, Indonesia

**Keywords:** caring, climate action, coping, disaster, family resilience, Islam, Pandemic, preparedness, stress

## Abstract

The study aimed to evaluate the longitudinal impact of ISTIFAR programme (Islamic-Based Training for Family Resilience) to the family state of coping, resilience and disaster preparedness on longitudinal observation. A quasi-experimental design was employed with pre- and post-test intervention group only. The sample size was 63 families that survived the earthquake, which sampled using purposive sampling. The sampling criteria were disaster survivor, head of household, Muslim and mentally healthy. The variable was pre- and post-observation of coping, family resilience and disaster preparedness, with confounding factor of stress. The interview performed by structured questionnaire. The data analysed with Mann–Whitney U test and ordinal regression (α < 0.05). The result was a statistical difference between the observation of 6 months prior and follow-up but a slight decline in all variables mean. In detail, coping (*p* = 0.000), family resilience (*p* = 0.000) and the disaster preparedness (*p* = 0.023). There was no statistical correlation between the coping, family resilience and the disaster preparedness towards pandemic stress (*p* = 0.747). Islamic-Based Training for Family Resilience positively impact coping, resilience and disaster preparedness, albeit slight declines over 6 months, but no evident correlation with coronavirus disease 2019 (COVID-19) pandemic stress.

**Contribution:**

This study contributes to the existing body of knowledge by highlighting the effectiveness of authentic disaster preparedness facilitated through ISTIFAR among vulnerable families. It suggests that enhancing resilience, particularly concerning disaster preparedness and, notably, amids the COVID-19 pandemic, can be achieved through authentic local methodologies. The grounded approach proves beneficial, indicating that interventions within communities should not be universally applicable but tailored to leverage local community wisdom.

## Introduction

The 2018 earthquake on Lombok Island affected approximately 417 529 inhabitants, 101 735 displaced with North Lombok Regency bearing the brunt of the devastation and claimed the lives of 537 people (Darmansyah, Sutisna & Widodo 2023; National Disaster Management Board 2018). Following the disaster, families faced social, economic and physical vulnerabilities exacerbated by limited healthcare access, economic upheaval because of agricultural land conversion and the lack of tourism (Darmansyah et al. 2023; Eko Jakandar 2020). Additionally, disasters negatively impact the mental health of the community and the people, which reduce the well-being (Bakic & Ajdukovic 2021). The range of mental health problems can vary from mild anxiety to serious PTSD (post-traumatic stress disorder), significantly reducing the ability to recover socially, economically and in disaster preparedness (Bakic & Ajdukovic 2021; Pandey 2019; Sellberg et al. 2018). The problem that this study addresses is a method to improve disaster survivors’ well-being by integrating spirituality alongside enhancing individual, interpersonal and community resources, acknowledging the religious context of the North Lombok community (Bakic & Ajdukovic 2021; McClintock et al. 2019).

The survivor of Lombok Island earthquake faced vulnerabilities such as limited healthcare access and economic instability, worsened by tourism-driven land conversion (Fitriadi et al. 2023; Ichsan & Waru 2019; Rejeki, Ilmiawan & Arif 2020). Vulnerable families are often unaware that their living environment is disaster prone because they depend on it for their livelihood. For instance, a study in the Bekkersdal mining area found that the community does not consider disaster risks because the majority work as miners (Madubula & Van Eeden 2024). However, family resilience against disasters is crucial for preparedness and recovery (Mayer 2019; Rose 2011). In North Lombok, one of the distinctive and prominent forms of social capital is the religious gatherings known as *tahlilan* and *yasinan* (Iwan et al. 2018). This demonstrates that the community’s religiosity is strong and may be the key to enhancing coping mechanisms, resilience and disaster preparedness (Febriani & Nurhayati 2021; Smith & Nichols 2020).

During the evaluation period of the ISTIFAR programme, the coronavirus disease 2019 (COVID-19) hit the area and potentially become a new stressor because the community was forced by the government to do social restrictions. The social restriction made the community unable to work, which then declined the family economy. The pandemic became a new stressor causing significant psychological distress and it tested the community adaptability. Especially among females, younger individuals and those with chronic illnesses or unemployed people (Xiong et al. 2020). Therefore, the purpose is to evaluate the longitudinal impact of ISTIFAR programme (Islamic-Based Training for Family Resilience) to the family state resilience, coping and disaster preparedness on longitudinal observation. The state of COVID-19 stress will also be investigated as the evaluation happens during the COVID-19 pandemic.

## Problem statement

The family affected by earthquake needs a good resilience for better recovery (Mayer 2019). However, the family is vulnerable to poor economic growth after the earthquake crippled their livelihood. The family showed low resilience, low coping and more alarmingly low disaster preparedness (Sriyono, Nursalam & Hamzah 2020). An interview with the community and the disaster relief volunteer found that the community needs approximately 2–3 years to fully recover economically and socially from devastating disaster. Social capital plays a crucial role in household economic recovery, with pre-disaster connections beneficial in the mid-term and post-disaster connections contributing to long-term recovery (Xiang, Welch & Liu 2021). The average time for asset recovery can be extensive, with factors such as education level, damage value and economic pressure influencing recovery duration (Sunarti et al. 2022).

Society needs methods for better recovery by enhancing resilience, with religion being a fundamental aspect of societal rules and values. Religious principles significantly influence one’s self-perception and emotional well-being (Miftahur & Hudriansyah 2022). In Indonesia, where the majority of the population is Muslim, particularly in West Nusa Tenggara, where 96% identify as Muslim (Amelia, Rahmah & Harahap 2021; BPS NTB 2018), religion plays a crucial role in overcoming challenges. Families employing effective religious coping mechanisms have a greater likelihood of achieving resilience compared to those with insufficient religious coping (Prasetyo et al. 2022). These coping strategies can be implemented not only at the societal level but also at the micro or family level for managing the disaster impacts.

In addition, during the time the research was conducted, COVID-19 was ongoing, and between the end of 2019 and December 18, 2020, it had infected 643,508 people in Indonesia. Approximately 86–97 confirmed cases, four deaths were reported, and hundreds were potentially infected in the region (Dinas Kesehatan NTB 2023; Pemprov NTB 2020; World Health Organization 2023). However, the proximity of the COVID-19 cases to the research location could not be confirmed. The government is using Large-Scale Social Restriction, which restrict an area’s space for movement, as a strategy to break the COVID-19 chain (Nasruddin & Haq 2020). The COVID-19 pandemic has complicated effects on a family’s social and economic well-being, which represent the smallest unit of a nation. Therefore, family members must develop and adapt in the face of adversity (Conger & Conger 2002; Walsh 2013).

## Research methods and design

### Research design

A quasi-experimental design was employed with pre- and post-test intervention group only. The study investigated the change in family resilience, coping and disaster preparedness after surviving earthquake. The family joined a designed family resilience training, which consist of resilience, coping and disaster preparedness improvement. Additionally, since the study was conducted during the COVID-19 pandemic, it also examined whether the stress caused by COVID-19 affected family resilience, coping, and disaster preparedness. The first hypothesis stated that there is significant difference between family resilience, coping and disaster preparedness after the ISTIFAR training. According to the second hypothesis, there is a significant correlation between the family resilience, coping and disaster preparedness to the COVID-19 pandemic stress.

### Sampling

The research population was vulnerable families in Medana Village, North Lombok, which survived the 2018 earthquake. The eligibility criteria were as follows: inclusion criteria were head of household (regardless of husband or wife) and Muslim. Exclusion criteria were non-resident of the area. The sampling technique used was purposive sampling and resulted in 63 families. The family is a unit so that the head of the family becomes the representative.

### Variable

The investigated variables were family resilience, coping and disaster preparedness. Those variables measured twice on pre- and post-test. Furthermore, the COVID-19 stress and preparedness measured to understand the current phenomenon that happens in the community. The family resilience measured in ordinal data of 5 scale according to Walsh family resilience phase: survival, adjustment, acceptance, growing stronger and helping others, respectively, from the lowest to the highest phase. The coping, disaster preparedness and COVID-19 pandemic stress measured ordinally by scale of low-, medium- and high. The COVID-19 preparedness reported in the percentage of people applies it.

### Intervention and instruments

The intervention was a resilience training for family named ISTIFAR programme. This training combined family resilience empowerment and Islamic caring. The aim of the training is to improve the family resilience, coping and the disaster preparedness. The training was designed for 1-month programme, which consisted of resilience training, coping strategy, disaster preparedness training, which was combined with the Islamic way. Every week, there were two sessions where material was presented to the families. The families were then assigned to apply what they learned in their daily lives. Islamic-Based Training for Family Resilience instructors evaluated the changes in family resilience throughout the programme. The instruments for interviewing and observing the family had been proven valid and reliable. The family resilience questionnaire came from the combination of Walsh family resilience questionnaire and Lietz family resilience process (*p* = 0.932 [α > 0.06]) (Lietz et al. 2016; Walsh 2016). The coping questionnaire adopted and modified from Ways of Coping Questionnaire (WCQ) (*p* = 0.978 [α > 0.6]) (Folkman 1988). The disaster preparedness questionnaire was developed by the research team reliability *p* = 0.845 (α > 0.6). In addition, the pandemic stress was measured using CoPaQ (The COVID-19 Pandemic Mental Health Questionnaire), in which already published and tested. The COVID-19 preparedness following the government recommendation (Rek et al. 2020).

### Data analysis

Firstly, descriptive data analyses were performed that described the frequency, mean and modes of the data collected. Normality and homogeneity test were also carried out to fulfil the assumption to conduct inferential analysis. Secondly, inferential analysis was performed by comparing the pre-test and post-test (the 6-month follow-up observation). Thirdly, the correlation between family resilience, coping and the disaster preparedness towards pandemic stress was analysed using ordinal regression.

### Ethical considerations

Ethical clearance to conduct this study was obtained from the Universitas Airlangga, Faculty of Nursing (no. 1882- KEPK).

## Result

The research was conducted from August to September 2020 in Medana Village, North Lombok Regency, West Nusa Tenggara, Indonesia. All of them were Muslim (Table 1).

**TABLE 1:** The demographic data of respondents.

Characteristics	Frequency	%	Mean or mode
**Age (years)†**	-	-	41.22
21–30	15	23	-
31–40	17	27	-
41–50	14	23	-
> 50	17	27	-
**Gender**	-	-	Male
Male	58	92	-
Female	5	8	-
**Education**	-	-	Elementary
Unschooled	16	25	-
Elementary	18	28	-
Middle high	11	17	-
Senior high	15	24	-
Higher educated	3	5	-
**Occupancy**	-	-	Worker
Health worker	2	3	-
Government staff	3	5	-
Restaurant, Grocery	10	16	-
Sanitary Officer	1	1	-
Worker	26	41	-
Housewife	2	3	-
Chief of Neighbourhood	1	1	-
Private sector	8	12	-
Merchant	2	3	-
Unemployed	8	12	-
**Salary (in IDR)**	-	-	≤ 1 million
≤ 1 million	33	52	-
> 1–2 million	19	30	-
> 2–3 million	5	8	-
> 3–4 million	6	10	-
**Government assistance**	-	-	Groceries assistance
Do not received any	14	22	-
Groceries assistance	20	33	-
Money assistance	15	23	-
Groceries and money assistance	14	22	-
**Health insurance**	-	-	BPJS
Self-financed	1	1	-
BPJS	62	99	-
**Islam prayer**	-	-	Always
Rarely	7	12	-
Often	28	44	-
Always	28	44	-

SD, standard deviation; IDR, Indonesian Rupiah; BPJS, Indonesian Government Health Insurance.

† , Age: standard deviation = 12.428.

**FIGURE 1:**
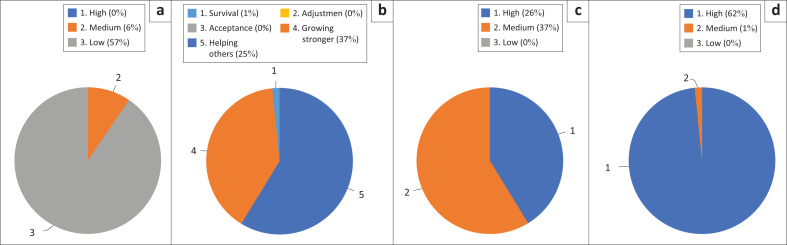
Statistic of: (a) pandemic stress (b) family resilience (c) coping (d) and disaster preparedness.

The doughnut diagram (Figure 1) illustrates stress levels, family resilience, coping and COVID-19 risk reduction. Most respondents reported low pandemic stress (90%), with the majority showing strong family resilience (59% in ‘Growing Stronger’). Coping levels were predominantly high. Nearly all respondents demonstrated high COVID-19 risk reduction, with only one at a moderate level. The majority of the north Lombok community has prepared for the COVID-19 pandemic as ordered by the government (Table 2).

**TABLE 2:** The coronavirus disease 2019 pandemic risk reduction.

Number	Risk reduction action	%
1.	Wearing mask	94
2.	Hand wash regularly	94
3.	Carrying liquid hand sanitiser everywhere	87
4.	Providing water loo in the front door	94
5.	Maintain physical distancing	98
6.	Avoid travel and vacation	95
7.	Avoid hand shaking	95
8.	Avoid hanging out	98
9.	Providing detergent, alcohol for disinfection	98
10.	Helping others buy groceries	98

### The evaluation of family resilience, coping and disaster preparedness

Comparison of pre- and post-test results showed significant differences in family resilience, coping and disaster preparedness, with values as follows: family resilience *p* = 0.000, coping *p* = 0.000 (α < 0.05) and the disaster preparedness *p* = 0.023 (α < 0.05). So that there is a decrease in family resilience, coping and disaster preparedness. There was an average decrease in the three variables, in detail as follows. The level of family resilience decreased by 11.87% (standard deviation [SD] ± 8.139), the coping decreased by −14.71% (SD ± 4.323) and the disaster preparedness −3.30 % (SD ± 4.085). The conclusion is a decrease (in %) of the level of family resilience, coping and disaster preparedness (Table 3).

**TABLE 3:** Evaluation of family resilience, coping and disaster preparedness 6 months post ISTIFAR program.

Variable	Δ (Mean difference pilot – follow up)			Mann-Whitney *U* test		Independent *T* test‡ (two-tailed)		
	Δ	SD	Δ (%)§	*Z* score	*p*	*T* score	*df*	*p*
Family resilience	−8.70	8.139	−11.87	−7.218	0.000	8.181	124	0.000
Coping	−5.16	4.323	−14.71	−7.298	0.000	9.881	124	0.000
Disaster preparedness → COVID-19 risk reduction	−2.06	4.085	−3.30	−2.601	0.009	2.848	124	0.023

SD, standard deviation; *df*, degree of freedom.

† , Not corrected for ties;

‡ , T table = 2.024;

§ , The ‘– Δ’ means the variable level is declining.

### The correlation of family resilience, coping and the disaster preparedness towards the coronavirus disease 2019 pandemic stress

The pandemic stress identification showed that the majority of respondents (90% [*n* = 57]) were at low levels. None of the respondents had high stress levels. The ordinal regression found that there was no significant correlation between the family resilience, coping and the disaster preparedness to the pandemic stress *p* = 0.747 (α < 0.05). This means the family resilience, coping and the disaster preparedness did not affect the stress that emerges to the community caused by the COVID-19 pandemic (Table 4).

**TABLE 4:** The correlation by ordinal regression.

Variable	Category	*n*	%	Minimum	Maximum	Mean	SD	Ordinal regression	
								*R* ^2^	*p*
Pandemic stress	High	0	0	-	-	-	-	-	-
	Moderate	6	10	-	-	-	-	-	-
	Low	57	90	0	27	12.22	-	-	-

	**Total**	**26**	**100**	**-**	**-**	**-**	**6.264**	**0.502**	**0.747**

Note: Scoring: Low = 0–19; Medium = 20–39; High = 40–56.

SD, standard deviation.

## Discussion

This study evaluates the longitudinal effects of ISTIFAR programme on earthquake-surviving families, 6 months post-intervention. The community’s ability to become resilient needs to be tested in real practice, where the practice of making a resilient community requires planning that is adaptive, collaborative and clarifies resource management goals (Mayer 2019; Sellberg et al. 2018). Given the concurrent non-natural disaster, the COVID-19 pandemic, this research is crucial in assessing ISTIFAR programme’s efficacy. Islamic-Based Training for Family Resilience programme is Islamic-focused approach, emphasising prayer and Quranic reading for spiritual enlightenment and surrender. The participants demonstrated increased levels of family resilience, coping, and disaster preparedness, with the majority classified as high. However, the research found decreased family resilience, coping and disaster preparedness during the subsequent evaluation coinciding with the pandemic, caused by adapting to lifestyle changes, especially Large-Scale Social Restriction in West Nusa Tenggara. The study’s findings are consistent with Özmete, who found that lockdown caused family function and resilience to decline during the COVID-19 pandemic (Özmete & Pak 2023). One family regressed to the survival phase of resilience, while coping improved for nine families. On an average, there was a decline of 9% in family resilience, 8% in coping and 6% in disaster preparedness across families. Sustaining community resilience gains is crucial, necessitating the development of adaptable, authentic community partnerships and collaboration to support communities amid evolving challenges such as COVID-19 (Liu et al. 2018; Pandey 2019).

Lietz proposes that family resilience is not a linear progression but can involve phase shifts, either increasing or decreasing (Lietz 2007). Accelerators and buffers influence these shifts, such as Islamic caring, encompassing faith, patience, gratitude, sincerity, virtuous actions and community ties. Integrating Islamic principles as the community influencing factors into family resilience training aims to expedite phase transitions, reinforcing families both structurally and emotionally (Lietz 2011; Slemp et al. 2020). Despite resilience’s potential decline amid adaptation, Islamic caring serves as both an accelerator and a safeguard, which is evident in its role in slowing down resilience erosion post-disaster. However, the study observed a slight decline in family resilience 6 months later because of the fact that the ISTIFAR programme only focused on post-earthquake cases, so it was necessary to adjust the topic about coping the COVID-19 pandemic. The community will assist from ISTIFAR programme, if it is modified to incorporate COVID-19 material, in terms of psychological strain and health issues that individuals experience as a result of the intense discourse on spiritual and religious principles (Tjahjadi et al. 2023; Upenieks 2022). The restrictions during the COVID-19 pandemic led to personality changes in respondents, with many shifting from helping others to focusing on survival, likely due to income loss (Irzalinda & Sofia 2019; Köhne, Engert & Rosendahl 2023). Uneven resilience interventions may lead to conspicuous resilience, potentially fostering social decline because of inequalities and power imbalances, reinforcing private interests (Gray 2023; Pandey 2019).

The family resilience, coping and the disaster preparedness do not affect the level of stress. However, the study found that families that practice worship regularly are more likely to have low stress level (Alghamdi et al. 2022; Islamia 2023). Most respondents exhibit low stress levels, with some reporting moderate stress, suggesting significant impact from the COVID-19 pandemic, especially among vulnerable populations (Tang & Li 2021). Economic challenges, exacerbated by inadequate government aid, heavily affect families (Polizzi, Lynn & Perry 2020). However, basic food assistance and government aid alleviate the economic strain for many (Knearem, Jo & Carroll 2021; Ogundari, Aromolaran & Akinwehinmi 2022; Susantyo et al. 2023). Additionally, enhanced family spirituality following ISTIFAR programme contributes to stress reduction (Smith & Nichols 2020). While the correlation between psychology and theology is not robust, theology supports psychological well-being, evidenced by improved family worship routines (Papaleontiou - Louca 2021). Thus, the study underscores the importance of spirituality in mitigating stress levels, particularly in times of crisis such as the COVID-19 pandemic (McClintock et al. 2019; Othman & Sipon 2012).

## Conclusion

The ISTIFAR programme demonstrated significant improvements in family resilience, coping and disaster preparedness immediately post-intervention. However, a longitudinal assessment revealed a decline in these areas, likely influenced by the COVID-19 pandemic and related lifestyle changes, such as Large-Scale Social Restrictions in West Nusa Tenggara. The study found no significant correlation between these variables and pandemic-related stress, suggesting that external stressors had a limited impact on family resilience and coping mechanisms. Enhanced spiritual practices among participants were associated with lower stress levels, underscoring the role of spirituality in maintaining psychological well-being during crises.
